# The utility of fluoroscopic venography during arteriovenous access creation for hemodialysis: A case report

**DOI:** 10.1097/MD.0000000000043727

**Published:** 2025-08-08

**Authors:** Yejin Jo, Sangho Lee, Suehyun Park, Hyung-Kee Kim, Seung Huh, Deokbi Hwang

**Affiliations:** a Department of Surgery, Division of Vascular and Endovascular Surgery, Kyungpook National University Hospital, Daegu, Republic of Korea; b Department of Surgery, Division of Vascular and Endovascular Surgery, Kyungpook National University Chilgok Hospital, Daegu, Republic of Korea; c Department of Surgery, School of Medicine, Kyungpook National University, Daegu, Republic of Korea.

**Keywords:** arteriovenous (AV) access, fluoroscopic venography, preoperative evaluation, ultrasound, vascular access

## Abstract

**Rationale:**

As the end-stage kidney disease (ESKD) population requiring vascular access for hemodialysis continues to grow, the Kidney Disease Outcomes Quality Initiative guidelines emphasize vessel preservation to ensure viability for future access. Preoperative ultrasound is commonly performed prior to arteriovenous (AV) access creation when available. However, its major limitation is the restricted imaging range, which impedes visualization of the complete vascular network. This study highlights the importance of reconsidering fluoroscopic venography as a complementary tool to ultrasound for improving AV access planning.

**Patient concerns:**

A man in his late forties and a septuagenarian woman with ESKD underwent AV access creation surgery for ongoing hemodialysis.

**Diagnoses:**

The surgical plans could not be determined solely based on preoperative ultrasound findings. In Patient 1, AV access using an artificial graft with the brachial artery as the inflow and the brachial vein as the outflow in a forearm loop configuration was considered as the primary option. In Patient 2, the cephalic vein drainage was unclear with ultrasound due to the clavicle.

**Interventions:**

The operation was performed with a transverse incision just below the elbow. Before the anastomosis, we conducted intraoperative fluoroscopic venography through the surgically exposed median cubital vein using a small amount of diluted contrast media to assess the overall venous drainage system in the upper arm.

**Outcomes:**

The initial surgical plans based on ultrasound findings were modified through intraoperative fluoroscopic venography. In Patient 1, the distal anastomosis was redirected to the median cubital vein, thereby preserving the deep vein. In Patient 2, fluoroscopic venography enabled the successful creation of AV access using an autologous vein instead of a graft by directly visualizing the cephalic vein drainage. At early follow-up, both accesses achieved successful maturation; however, long-term outcomes could not be fully assessed, and no access-related complications were observed during the observation period.

**Lessons:**

Intraoperative fluoroscopic venography allowed for more precise AV access planning by providing real-time visualization of venous anatomy. This approach can facilitate intraoperative decision-making and help expand access options for ESKD patients while preserving future options.

## 1. Introduction

The prevalence of end-stage kidney disease (ESKD) requiring kidney replacement therapy has risen dramatically due to aging populations and increasing metabolic disorders.^[1–3]^ Among kidney replacement therapy options, hemodialysis is the predominant modality, leading to a high demand for vascular access (VA).^[4,5]^ Selecting the appropriate VA (autologous fistula, graft, or central venous catheter) is critical for effective hemodialysis over a patient’s lifetime. The most recent Kidney Disease Outcomes Quality Initiative guidelines have shifted their emphasis from the traditional fistula first approach to prioritizing the preservation of future VA options.^[6]^ This underscores the critical need for accurate and comprehensive vessel evaluation prior to arteriovenous (AV) access creation, particularly in patients with complex venous anatomy. Duplex ultrasound has become the primary imaging tool for evaluating vessels prior to the creation or revision of AV access, owing to its noninvasiveness, lack of nephrotoxic materials, acceptable accuracy, reproducibility, and potential for concurrent hemodynamic assessment.^[7,8]^ However, its limitations, including operator dependency and limited field of view, often require supplementation with other imaging modalities. Angiography, once the gold standard for vascular diagnosis, is now less favored due to its invasiveness and nephrotoxic risks.^[9–11]^ Employing fluoroscopic venography in conjunction with ultrasound, while mitigating its drawbacks, could enhance the precision of evaluations for AV access creation. This report presents 2 cases where fluoroscopic venography, performed through surgically exposed veins, provided critical information for AV access creation when ultrasound alone was insufficient. Approval for this study was granted by our institutional review board (IRB #2024-11-027), and written informed consent was obtained from both patients.

## 2. Case presentation

### 2.1. Case 1

**Figure 1.**
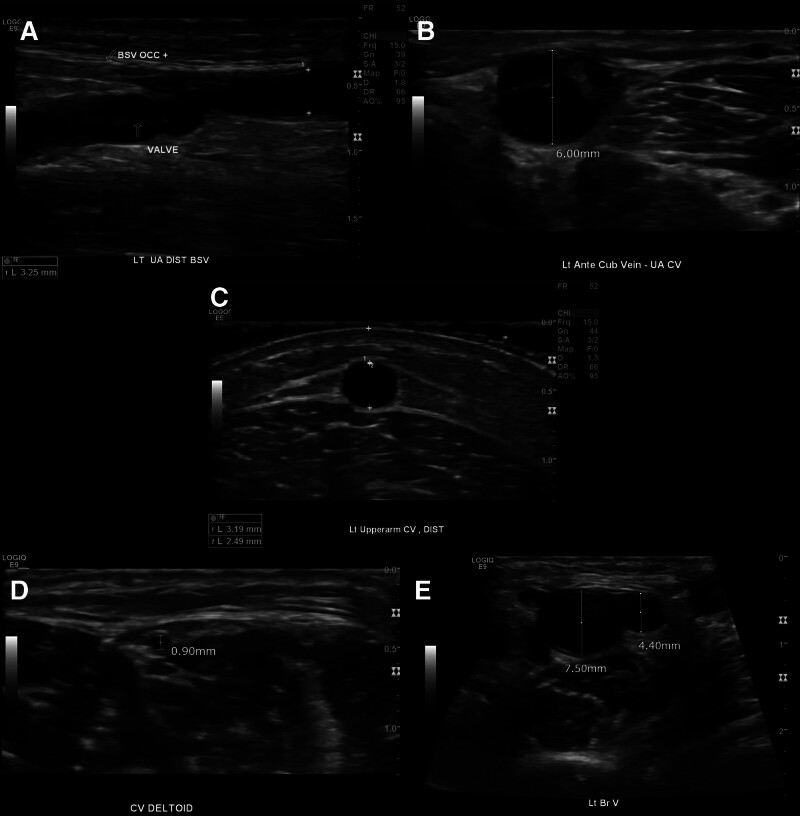
Preoperative ultrasonography findings in Patient 1. The vein quality was generally poor within the observable range. (A) The basilic vein in the upper arm displayed a hyperplastic wall with valves and partial occlusion. (B) The median cubital vein had a diameter of 6 mm and a patent lumen, although it exhibited a partially hyperplastic wall. (C, D) The cephalic vein in the upper arm was in good condition, but its diameter sharply decreased to 0.9 mm at the deltoid level. (E) The brachial vein exhibited good quality with a diameter exceeding 4 mm, and patency was maintained up to the axillary vein.

A 49-year-old male visited our clinic in 2023 for the creation of AV access. He had a history of splenectomy due to idiopathic thrombocytopenic purpura 30 years prior and had received a kidney transplantation (KT) from his wife 13 years earlier. Two years before and 1 year after the KT, the patient underwent creation and ligation of a radio-cephalic arteriovenous fistula (AVF) respectively. Thirteen years post-KT, he required permanent AV access creation for hemodialysis due to renal function deterioration from acute antibody-mediated rejection. Since the KT, he had been taking mycophenolate mofetil 500 mg in the morning and 250 mg in the evening, tacrolimus 1 mg twice daily, and prednisolone 5 mg once daily. Admission laboratory results are presented in Table 1. Notably, thrombocytopenia (platelet count 75 × 10^3^/µL) and marked azotemia (blood urea nitrogen [BUN] 109.3 mg/dL) were observed. Echocardiography revealed a normal ejection fraction (66%) and no regional wall motion abnormalities. On physical examination, no prominent superficial veins were palpable. Preoperative duplex ultrasound (Fig. 1) indicated poor vein quality in the forearm. The basilic vein wall appeared diffusely hyperplastic, and the cephalic vein post-radio-cephalic anastomosis was hyperplastic and nearly occluded with chronic thrombosis. The upper arm veins were superior to those in the forearm but still insufficient for autologous AV access creation. The basilic vein exhibited hyperplastic wall and valves, while the cephalic vein in the deltopectoral groove measured 0.9 mm in diameter. Despite thickening potentially due to multiple vein punctures, the median cubital vein had an adequate diameter of 6 mm and multiple patent branches. However, these branches ran deep through the muscles and drained solely into the upper arm cephalic vein within the observable range. Due to its depth, the cephalic vein was untraceable under the deltoid muscle using duplex ultrasound. The brachial vein displayed good quality with a diameter exceeding 4 mm, maintaining patency up to the axillary vein. The central vein was presumed patent, suggested by the positive respiratory augmentation of the subclavian vein. The brachial artery’s quality was also favorable without any calcification. Consequently, AV access using an artificial graft with the brachial artery as the inflow and the brachial vein as the outflow in a forearm loop configuration was considered as the primary option.

**Table 1:** Admission (preoperative) laboratory findings of Patients 1 and 2.

Parameter	Ref. range	Patient 1	Patient 2
WBC (×10³/µL)	4.0–10.0	6.46	6.60
Hemoglobin (g/dL)	12–16	10.5	12.1
Platelet (×10³/µL)	150–400	75↓	163
AST (U/L)	0–40	16	20
ALT (U/L)	0–40	7	13
Creatinine (mg/dL)	0.6–1.3	3.57↑	5.39↑
BUN (mg/dL)	6.0–20.0	109.3↑	33.0↑
Potassium (mmol/L)	3.4–4.9	4.6	4.0
Calcium (mg/dL)	8.6–10.2	8.7	NA
Phosphorus (mg/dL)	2.5–4.5	4.5	NA
Total CO_2_ (mmol/L)	22–29	20.2	28.6

NA = not available.

**Figure 2.**
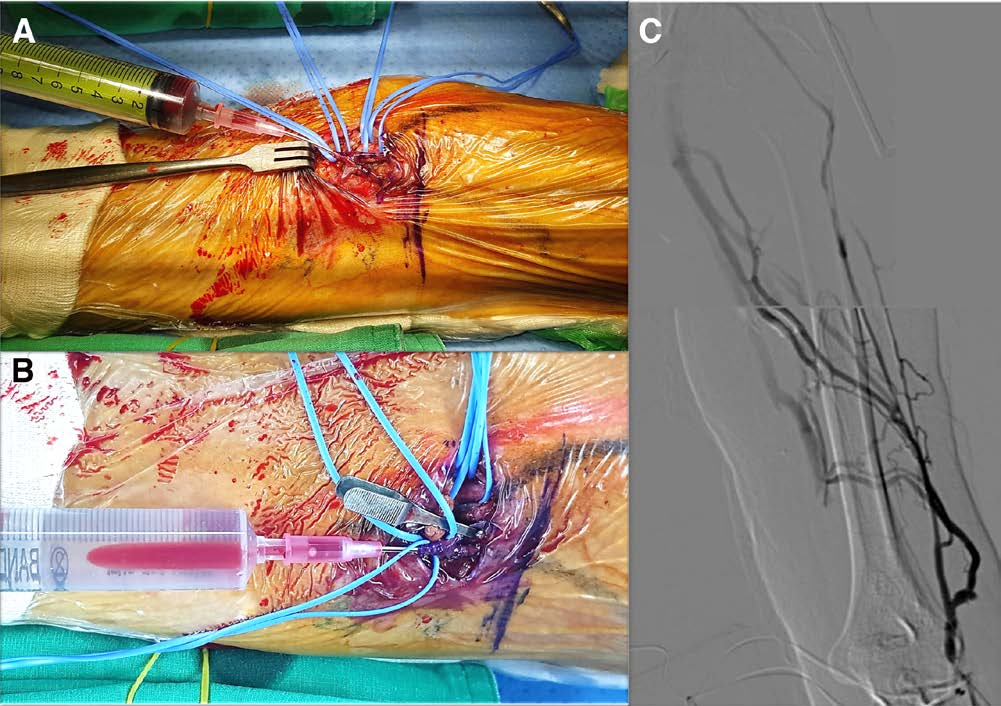
Operation field for intraoperative fluoroscopic venography and the fluoroscopic venography findings in Patient 1. (A) To assess the cephalic vein drainage, we punctured the surgically exposed median cubital vein with a 20-gauge angiocatheter and performed fluoroscopic venography using <20 cc of a 1:1 mixture of iodixanol contrast media (Visipaque®) and saline. (B) To concentrate cephalic vein drainage, the median basilic vein was clamped with a vein bulldog. (C) The fluoroscopic venography through the surgically exposed median cubital vein demonstrated that the flow was effectively drained into the deep veins, including the brachial, axillary, and subclavian veins via numerous intramuscular veins, even though the cephalic vein tapered toward a more proximal level.

Under local anesthesia, 10 mL of 1% lidocaine with epinephrine 1:100,000 was infiltrated subcutaneously along the transverse incision just below the elbow. Before the anastomosis, we conducted intraoperative fluoroscopic venography through the surgically exposed median cubital vein to assess the drainage of the overall vein system in the upper arm (Fig. 2A and B). The fluoroscopic venography demonstrated that the median cubital vein effectively drained into the deep brachial, axillary, and subclavian veins through intramuscular veins, although the cephalic vein narrowed towards a more proximal level (Fig. 2C).

Based on the venography results, we opted for a venous anastomosis at the median cubital vein rather than the brachial vein. Consequently, we created a left brachio-antecubital AV graft in a forearm loop configuration. No complications, such as arm swelling or graft occlusion, were noted during more than 2 years of follow-up after AV access creation. Maintenance hemodialysis was initiated via the AV graft 10 months after its creation. Using an FX 60 high-flux polysulfone dialyzer, treatments were performed 3 times weekly for 4 hours each at blood- and dialysate-flow rates of 200 mL/min and 500 mL/min, respectively; net ultrafiltration was 1.5 to 2.0 L. After 1 month, potassium fell from 5.7 to 4.9 mmol/L and BUN from 111.4 to 81.4 mg/dL, but adequacy remained low, with a single-pool Kt/V (spKt/V) of approximately 0.4, so the prescription is being intensified. No intradialytic adverse events have occurred. Meanwhile, immunosuppressants were discontinued 1 month after dialysis initiation, and prednisolone was tapered over the next 2 months and then stopped; no access-related complications occurred during or after this adjustment.

### 2.2. Case 2

**Figure 3.**
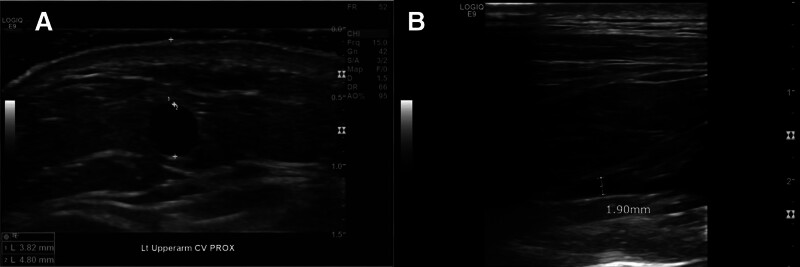
Preoperative ultrasound findings in Patient 2. (A) The upper arm cephalic vein had a suitable diameter (>4 mm) for creating autologous AV access, but (B) the drainage pathway from the arch to the subclavian vein was obscured by the clavicle.

A 71-year-old female with a history of hypertension, diabetes, kyphosis, and dementia presented at our outpatient clinic for a consultation regarding permanent AV access. She had been undergoing hemodialysis via a tunneled cuffed catheter on the right internal jugular vein for the past 3 months. She was referred to our center for elective creation of a permanent AV access, allowing removal of the catheter and secure long-term hemodialysis. On admission, azotemia (BUN 33.0 mg/dL) and elevated creatinine (5.39 mg/dL) were noted (Table 1). Echocardiography indicated normal heart function (ejection fraction 65%). Preoperative duplex ultrasound revealed that the diameters of the forearm veins were too small for AVF creation, yet the upper arm cephalic vein from the median cubital vein was deemed suitable for autologous AV access creation. However, the drainage of the cephalic vein was ambiguous, particularly from the arch to the subclavian vein. The confluence with the subclavian vein at the arch vein was not clearly delineated due to the clavicle (Fig. 3). The surgical plan remained undecided because the cephalic vein drainage was unclear. After confirming the arch vein with intraoperative fluoroscopic venography, we planned to create an autologous AV access if the cephalic vein drainage was adequate, or to use an artificial graft if inadequate.

**Figure 4.**
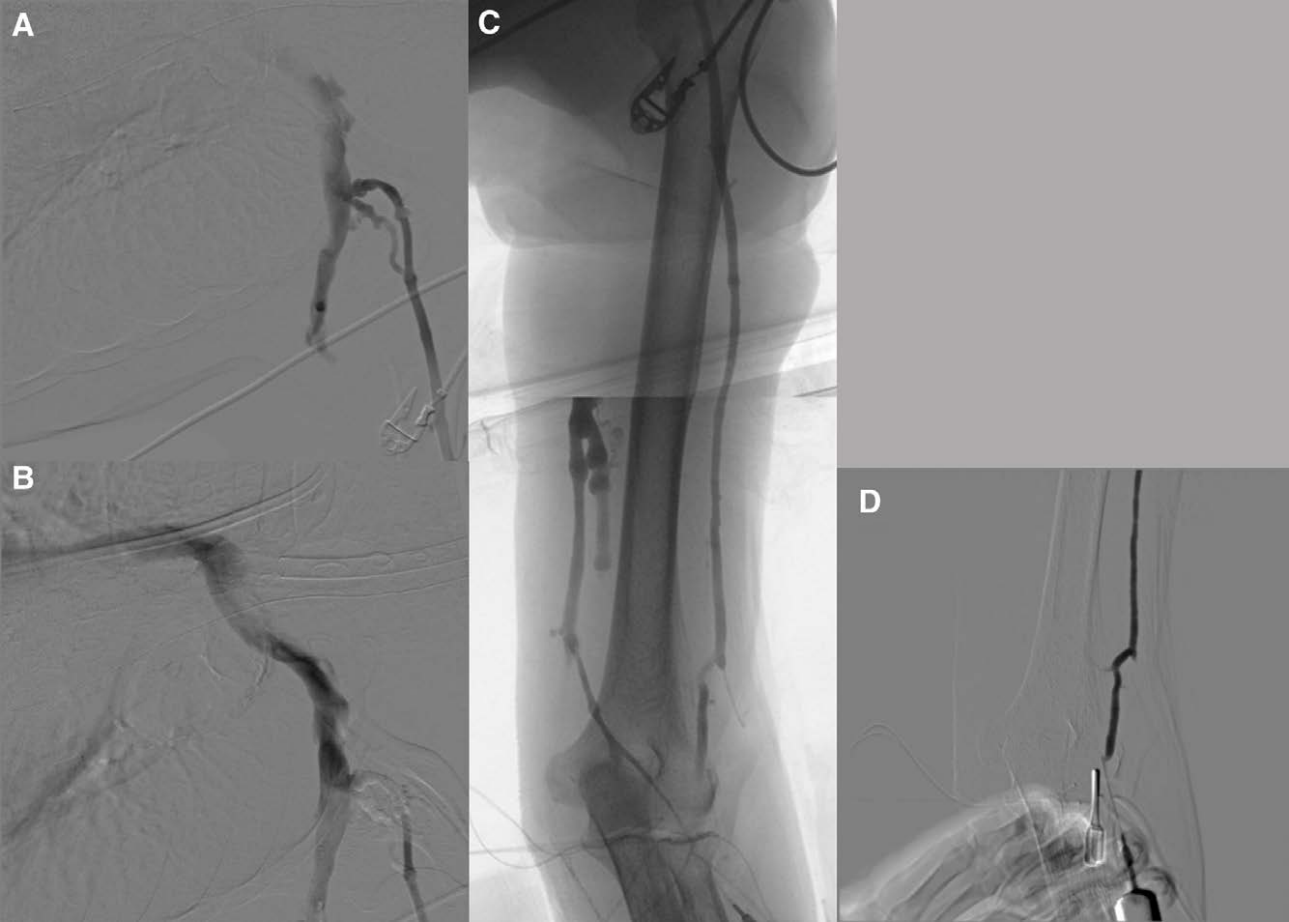
Intraoperative venographic findings in Patient 2. (A, B) The cephalic vein arch and subclavian vein exhibited normal flow without significant stenosis or external compression. (C, D) The overall diameter and course of the upper arm cephalic vein were appropriate for autologous AV access creation.

**Figure 5.**
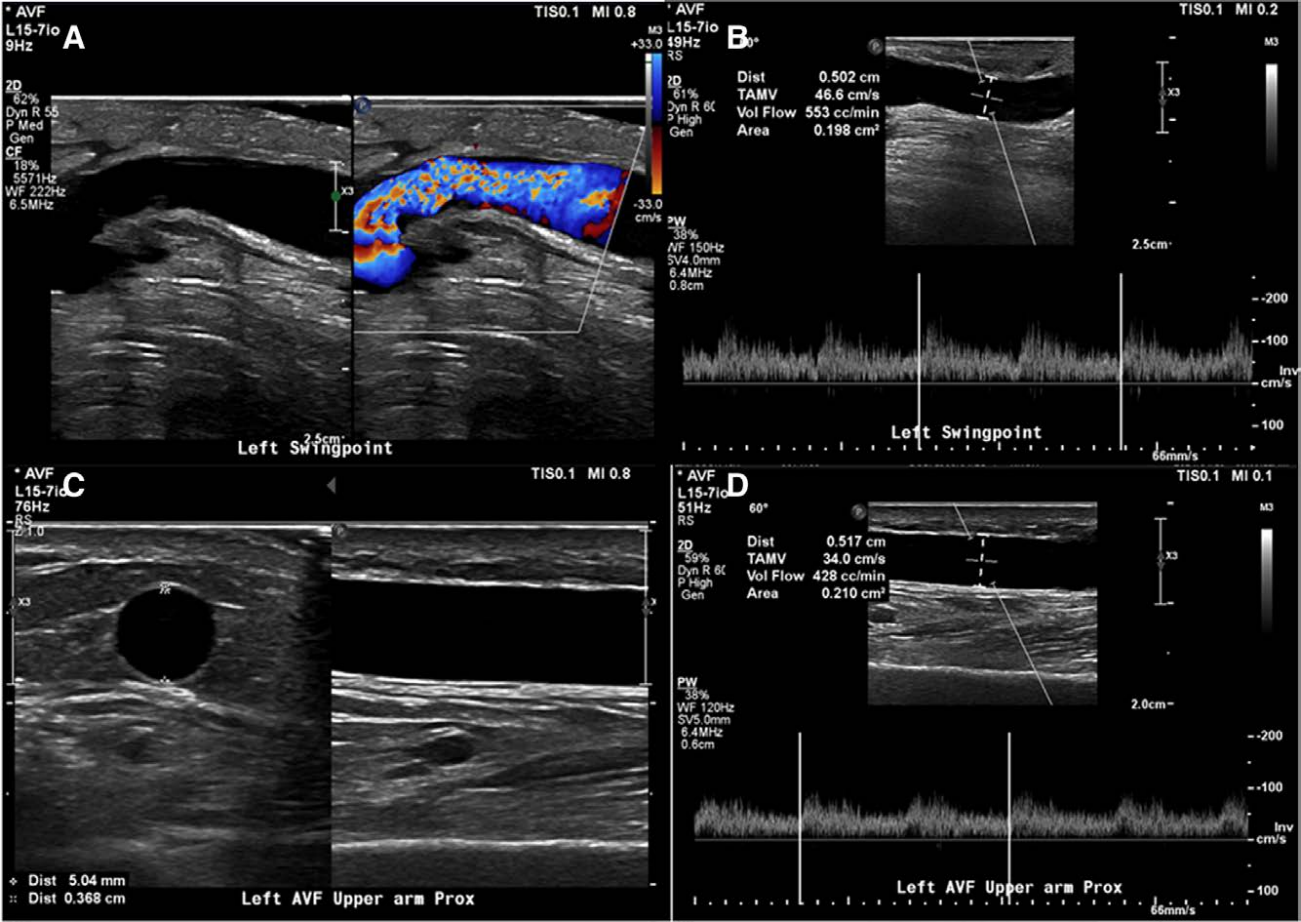
Two-month postoperative duplex ultrasound findings in Patient 2. (A) No stenosis is seen from the brachial artery-cephalic vein anastomosis to the contiguous cephalic vein segment. (B) At the swing point of the cephalic vein, the volume flow is 550 mL/min and the luminal diameter is approximately 5 mm. (C, D) In the proximal upper arm segment, the cephalic vein also measures approximately 5 mm in diameter, with a volume flow of 428 mL/min.

Given the patient’s diminished cooperation due to dementia, the operation was performed under general anesthesia. To assess the cephalic vein drainage, we punctured the surgically exposed median cubital vein with a 20-gauge angiocatheter and performed fluoroscopic venography using <20 cc of a 1:1 mixture of iodixanol contrast media (Visipaque®) and saline. Venography showed normal flow in the cephalic vein arch and subclavian vein without significant stenosis (Fig. 4). Consequently, we created a left brachio-cephalic AVF. Two months postoperation, the fistula’s maturation was confirmed via duplex ultrasound; the volume flow was 550 mL/min, and the cephalic vein’s diameter was approximately 5 mm (Fig. 5). The patient was referred back to her local dialysis center to initiate hemodialysis via the AVF and did not return for follow-up; consequently, late access-related complications or dialysis outcomes could not be evaluated.

## 3. Discussion

The latest version of the Kidney Disease Outcomes Quality Initiative guidelines emphasizes thoughtful planning for future AV access options tailored to an individual’s ESKD Life-Plan, shifting away from the strict adherence to the traditional fistula first strategy.^[6]^ To ensure viability for future access and to reduce VA-related complications, it is important to conduct a thorough evaluation of the vascular anatomy prior to surgery. Currently, preoperative ultrasound is almost universally performed when available, due to its benefits of noninvasiveness, repeatability, absence of nephrotoxicity concerns, and cost-effectiveness.^[7,8]^ Nonetheless, the primary limitation of ultrasound is its restricted imaging range, which hampers the visualization of the entire vascular network: particularly if obstructed by bony structures, calcifications, or deeply embedded in the muscle layer. This article presents 2 cases in which intraoperative fluoroscopic venography was used to overcome the limitations of ultrasound, resolving discrepancies between sonographic findings and actual venous drainage in each case.

In case #1, the surgical plan was altered to anastomose distally to the median cubital vein rather than the brachial vein, thereby preserving more options for future access by safeguarding the deep vein. In case #2, the definition of cephalic vein drainage was obscured by the clavicle on ultrasound. If the decision had been based solely on ultrasound, an artificial graft might have been used to create AV access. However, the use of fluoroscopic venography enabled successful creation of AV access with an autologous vein instead of a graft. Neither case required additional hemodialysis postoperatively, as only a small amount of diluted contrast media was used.

For several decades, conventional angiography using contrast media was regarded as the gold standard for assessing the location and severity of various cardiovascular pathologies.^[12–14]^ Although it has largely been supplanted by other noninvasive modalities such as duplex ultrasound, computed tomography angiography, or magnetic resonance angiography, it remains critically important in cases requiring immediate intervention following disease assessment or when noninvasive modalities yield ambiguous results. Regarding AV access creation, preoperative venography is now deemed reasonable only in cases of suspected central vein occlusion. For revisions, endovascular treatments such as percutaneous balloon angioplasty or stent-graft insertion under fluoroscopic guidance are recommended as primary modalities.^[6,15]^

Previously, it has been reported that identifying anatomical variants and occult veins is crucial for surgical planning and optimizing the outcomes of AV access creation.^[16,17]^ The literature indicates that venography may be beneficial for patients with suspicious physical findings related to central vein issues, or a history of catheterization, trauma, or surgery on the arm targeted for AV access.^[18]^ Although central vein occlusion was not suggested in our case, venography was useful in providing additional information about hidden anatomy by illustrating venous drainage in the arm designated as the access site. It can serve as a foundation for employing fluoroscopic venography in certain patients to refine the selection of AV access.

This article is significant in that it advocates for the reevaluation of fluoroscopic venography, which addresses the limitations of ultrasound without causing harm. Intraoperative fluoroscopic venography enabled the direct visualization of the draining venous system beyond the locational constraints, which are difficult to overcome with ultrasound alone. Consequently, future options are preserved by utilizing an autologous vein for AV access creation and performing distal anastomosis on a superficial vein rather than a deep one. Thus, it restored opportunities that might otherwise have been lost if decisions were based solely on ultrasound findings. Furthermore, inappropriate selection of the AV access type can result in significant socioeconomic burdens as well as physical and psychological exhaustion for patients. Therefore, ensuring the creation of an appropriate AV access in a single surgical procedure, with the supplementary use of fluoroscopic venography, is essential for minimizing such risks and optimizing long-term outcomes.

Several limitations associated with fluoroscopic venography can be acknowledged: (1) although fluoroscopic venography raises concerns about radiation exposure, its use in this study was limited to brief, single procedures for confirmation, significantly reducing the risk and making it a clinically acceptable option. (2) Although the use of contrast media is a limitation, in this study, fluoroscopic venography was performed with only 10 to 20 cc of contrast media, thereby mitigating concerns about contrast-induced nephrotoxicity.^[19]^ However, in cases where there is concern about declining renal function even with these small amounts, carbon dioxide can be used as an alternative contrast agent instead of iodinated contrast.^[20]^ (3) Since venography access is gained through the surgically exposed field for dissection of the brachial artery, additional incisions or punctures at other sites are unnecessary. Nonetheless, concerns remain that the invasiveness of intraoperative fluoroscopic venography is justified only when the vein within the surgically exposed field is utilized, limiting its usefulness primarily to cases where it is necessary to confirm the drainage of the median cubital vein or its nearby branches. However, the utility of other veins, such as the basilic vein, axillary vein, or cephalic vein of the forearm, seems certain with only ultrasound evaluation. (4) While fluoroscopic venography may incur higher initial costs compared to duplex ultrasound alone, its ability to provide detailed venous anatomy and improve procedural success rates may reduce overall healthcare costs in the long term. (5) Another limitation is that fluoroscopic venography cannot currently provide quantitative measurements of vascular resistance and drainage volume. However, the visualization of flow characteristics, such as flow speed, branch number, and caliber size, substantially addresses this limitation. Continued studies will be beneficial for a quantitative approach to better understand hemodynamics, including the speed and total volume of draining flow, thereby enhancing knowledge.

Although long-term follow-up was limited, both accesses achieved successful maturation and provided robust hemodialysis without complications, suggesting that intraoperative fluoroscopic venography is a safe and effective adjunct to ultrasound for optimizing AV access selection in certain patients.

## 4. Conclusion

The application of fluoroscopic venography via the surgically exposed median cubital vein during AV access creation provides a comprehensive view of the draining vein system, which could not be achieved with ultrasound alone. Thus, intraoperative fluoroscopic venography offers ESKD patients the optimal AV access option for hemodialysis while allowing for additional future options.

## Author contributions

**Conceptualization:** Deokbi Hwang.

**Data curation:** Yejin Jo, Sangho Lee, Deokbi Hwang.

**Formal analysis:** Yejin Jo, Deokbi Hwang.

**Investigation:** Suehyun Park, Deokbi Hwang.

**Methodology:** Deokbi Hwang.

**Supervision:** Hyung-Kee Kim, Seung Huh, Deokbi Hwang.

**Validation:** Hyung-Kee Kim, Seung Huh, Deokbi Hwang.

**Visualization:** Deokbi Hwang, Sangho Lee, Suehyun Park.

**Writing – original draft:** Yejin Jo, Deokbi Hwang.

**Writing – review & editing:** Sangho Lee, Suehyun Park, Hyung-Kee Kim, Seung Huh, Deokbi Hwang.
